# Spindle Orientation Regulation Is Governed by Redundant Cortical Mechanosensing and Shape-Sensing Mechanisms

**DOI:** 10.3390/ijms26125730

**Published:** 2025-06-15

**Authors:** Rania Hadjisavva, Paris A. Skourides

**Affiliations:** Department of Biological Sciences, University of Cyprus, University Avenue 1, New Campus, Nicosia 2109, Cyprus; rhadji05@ucy.ac.cy

**Keywords:** spindle orientation, focal adhesion kinase, mitosis

## Abstract

Spindle orientation (SO) plays a critical role in tissue morphogenesis, homeostasis, and tumorigenesis by ensuring accurate division plane positioning in response to intrinsic and extrinsic cues. While SO has been extensively linked to cell shape sensing and cortical forces, the interplay between shape- and force-sensing mechanisms remains poorly understood. Here, we reveal that SO is governed by two parallel mechanisms that ensure redundancy and adaptability in diverse cellular environments. Using live-cell imaging of cultured cells, we demonstrate that the long prometaphase axis (LPA) is a superior predictor of SO compared to the long interphase axis, reflecting adhesive geometry and force distribution efficiently at prometaphase. Importantly, we uncover a pivotal role for focal adhesion kinase (FAK) in mediating cortical mechanosensing to regulate SO in cells undergoing complete metaphase rounding. We show that in cells with complete metaphase rounding, FAK-dependent force sensing aligns the spindle with the major force vector, ensuring accurate division. Conversely, in cells retaining shape anisotropy during mitosis, a FAK-independent shape-sensing mechanism drives SO. These findings highlight a dual regulatory system for SO, where shape sensing and force sensing operate in parallel to maintain division plane fidelity, shedding light on the mechanisms that enable cells to adapt to diverse physical and mechanical environments.

## 1. Introduction

Mitotic spindle orientation (SO) is a highly regulated process that plays a fundamental role in embryonic development, tissue morphogenesis, and homeostasis in multicellular organisms. The orientation of the spindle determines the division plane and ensures the proper positioning of daughter cells, contributing to the establishment and maintenance of tissue architecture [1]. During embryogenesis, defects in SO lead to aberrant morphogenesis and organogenesis [2,3]. In adults, misregulated SO has been implicated in tumorigenesis and cancer progression [4,5].

In epithelial tissues, where cells typically divide symmetrically, the spindle aligns parallel to the tissue plane to ensure that daughter cells remain within the epithelium [6,7]. Misoriented divisions can lead to the detachment of daughter cells from the epithelial sheet, disrupting tissue organization. Additionally, SO contributes to the direction of tissue expansion. For instance, during Drosophila germ band elongation, planar SO aligns with the tissue’s elongation axis to facilitate directional growth, with defects causing structural abnormalities such as misshapen wings [8]. Similar mechanisms operate in Xenopus embryos, where oriented planar divisions are critical for processes like epiboly and neural tube formation [9,10,11].

While SO is essential for maintaining tissue architecture and homeostasis, it is also dynamically influenced by intrinsic and extrinsic cues. Intrinsically, the conserved Gαi/LGN/NuMA complex (Gαi/Pins/Mud in Drosophila) plays a central role by capturing astral microtubules at the cortex and aligning the spindle [12,13,14,15,16]. Extrinsic factors, such as cell geometry and mechanical forces, also regulate SO. Hertwig first proposed that the spindle aligns with the longest axis of the cell to divide it symmetrically, a principle supported by subsequent work. Minc et al. demonstrated that spindle alignment follows the longest axis of symmetry when cells are confined to pre-determined shapes [17], and O’Connell and Wang further suggested that astral microtubules mediate this shape-sensing mechanism by exerting pulling forces proportional to their length [18]. Furthermore, Strauss et al. showed that cell shape alone can dictate spindle orientation in polarized Xenopus blastomeres, driving asymmetric divisions and contributing to cell fate diversity during early development [19].

Mechanical forces at the cell cortex also play a critical role in SO. Studies by Théry et al. and Fink et al. using micropatterning techniques revealed that SO aligns with the major force vector, which reflects the long axis of anisotropic cell shapes [20,21,22]. Retraction fibers (RFs), remnants of interphase geometry, act as cortical tethers during mitosis, guiding the spindle along the primary force vector. Similarly, Petridou et al. showed that mechanical cues are transmitted through integrins at the lateral cortex, forming a cortical mechanosensory complex (CMC) that aligns the spindle along the greatest force vector, even in the absence of ligand binding [23].

Whether shape sensing and force sensing act independently or in concert to regulate SO remains unclear. Disentangling these mechanisms has been challenging, as cells often experience complex and overlapping cues in vivo, and the mechanism through which external forces are translated into spindle responses is still poorly understood. Recent work has suggested that NuMA responds to tissue-level forces directly, yet which upstream regulators are in control of this translocation is not clear [24].

Here, we investigate the interplay between shape sensing and force sensing on SO, focusing on the role of focal adhesion kinase (FAK) as a key mediator of force sensing. We demonstrate that these mechanisms act in parallel: shape sensing, driven by geometrical constraints, dominates when cells retain anisotropy during metaphase, whereas force sensing, mediated by FAK, becomes critical when cells undergo full metaphase rounding. Importantly, we show that prometaphase geometry is a better predictor of SO than interphase geometry in cells that achieve full rounding. This suggests that SO in cells that achieve full rounding is not determined via a memory of interphase geometry but rather by the force distribution around the cortex. Our findings highlight a robust, redundant system of SO regulation that ensures accurate division plane positioning under diverse mechanical and adhesive conditions.

## 2. Results

### 2.1. Geometric Anisotropy During Prometaphase Is a Better Predictor of SO than the Long Interphase Axis

**Figure 1:**
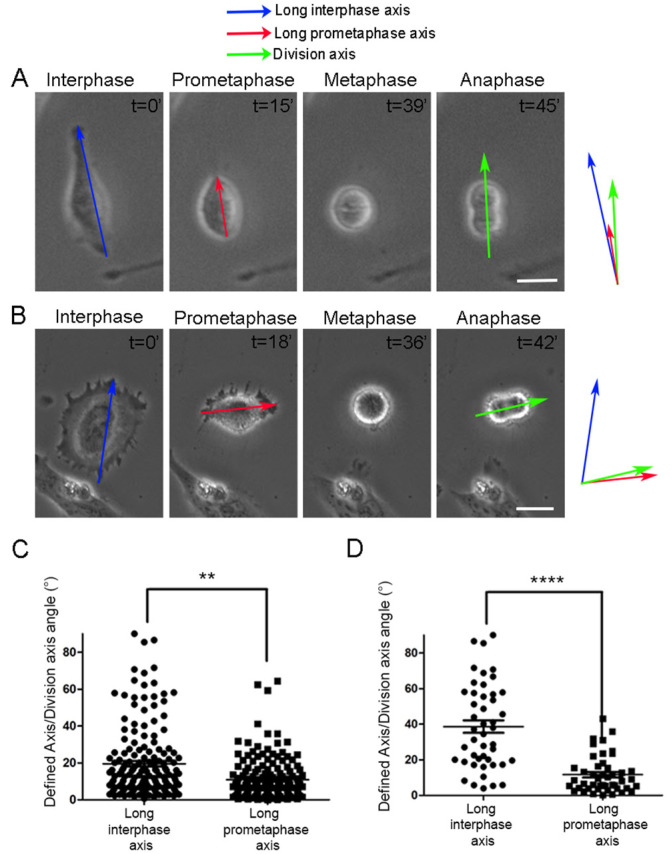
The long prometaphase axis is a more accurate predictor of the division plane in comparison with the long interphase axis in HeLa cells. (**A**) Series of still images from a live recording of a mitotic HeLa cell with a similar long interphase/long prometaphase axis. Blue arrow: long interphase axis, red arrow: long prometaphase axis, green arrow: division axis. t = time in minutes. The colors of arrows will be the same in all similar Figures. Scale bar = 10 μm. (**B**) Series of still images from a live recording of a mitotic HeLa cell with different long interphase/long prometaphase axes. Blue arrow: long interphase axis, red arrow: long prometaphase axis, green arrow: division axis. t = time in minutes. Scale bar = 10 μm. (**C**) Graph comparing the angle between the long interphase axis/division axis and the long prometaphase axis/division axis in HeLa cells. The angle measured indicates the degrees of difference between each axis with respect to the division plane. Means ± SEM: Long interphase axis: 13.41 ± 1.696 (*n* = 81), long prometaphase axis: 8.700 ± 1.179 (*n* = 82). Statistical analysis was carried out using a paired *t*-test, *p* = 0.0022 (**). *n*, number of cells measured from three independent experiments. (**D**) Graph comparing the angle between the long interphase axis/division axis and the long prometaphase axis/division axis in a subset of cells from graph (**C**) in which the difference between the long interphase and long prometaphase axis was more than 15 degrees. Means ± SEM: Long interphase axis: 42.51 ± 4.452 (*n* = 32), long prometaphase axis: 10.35 ± 1.524 (*n* = 32). Statistical analysis was carried out using a paired *t*-test, *p* ≤ 0.0001 (****). *n*, number of cells measured from three independent experiments.

**Figure 2:**
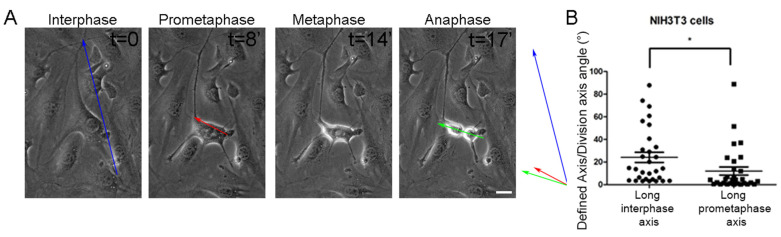
The long prometaphase axis accurately predicts the division plane in mouse embryonic NIH3T3 fibroblast cells. (**A**) Series of still images from a live recording of a mitotic NIH3T3 cell with different long interphase/long prometaphase axes. Blue arrow: long interphase axis, red arrow: long prometaphase axis, green arrow: division axis. t = time in minutes. Scale bar = 10 μm. (**B**) Graph comparing the angle between the long interphase axis/division axis and the long prometaphase axis/division axis in NIH3T3 cells. Means ± SEM: Long interphase axis: 24.14 ± 4.487 (*n* = 30), long prometaphase axis: 12.04 ± 3.567 (*n* = 30). Statistical analysis was carried out using a paired *t*-test, *p* ≤ 0.0116 (*). *n*, number of cells measured from three independent experiments.

Cell adhesion geometry can effectively predict the SO of adherent cells, which is typically parallel to the interphase long axis. Fink et al. previously showed that actin-based retraction fibers formed during the metaphase act as a footprint of interphase geometry and exert forces on the mitotic cortex, guiding SO along the major force vector, which corresponds to the long interphase axis due to the fact that longer RFs exert stronger forces [22] (Figure 1A). However, analysis of the time-lapse sequence of Hela cells revealed that, although the majority of cells orient their spindles parallel to the long interphase axis, a subset of cells fail to align with the interphase long axis, even if a clear long interphase axis is present before mitotic entry (Figure 1B). Careful analysis of the misoriented cell subpopulation revealed an inconsistency between the long interphase axis and the last axis of anisotropy prior to full cell rounding. This axis was typically established during the prometaphase due to variable rates of edge retraction. We termed this axis the long prometaphase axis (LPA), and extensive quantification of mitotic events showed that this axis is a more accurate predictor of SO than the long interphase axis in all cells (Figure 1C). When the difference between the two axes (long interphase and long prometaphase axis) was greater than 15 degrees, the long prometaphase axis was significantly more accurate in predicting SO (Figure 1D). We went on to test if the LPA is a more accurate predictor of SO in the mouse embryonic fibroblasts NIH/3T3 to examine whether this is conserved across different cell types. Live imaging of NIH/3T3 cells revealed that, similarly to HeLa cells, LPA is more accurate in the prediction of the division plane (Figure 2A,B). We propose that the long prometaphase axis reflects the adhesive strength around the cell, with areas that are better attached displaying delayed detachment during cell rounding. Cell areas that are strongly attached will establish denser retraction fibers and thus exert increased force on the mitotic cell cortex. As a result, prometaphase geometry provides a more accurate representation of force distribution around the mitotic cortex than interphase adhesion geometry and can, thus, more reliably predict SO.

### 2.2. FAK Null Cells Fail to Orient Along the Long Interphase and Long Prometaphase Axis

**Figure 3:**
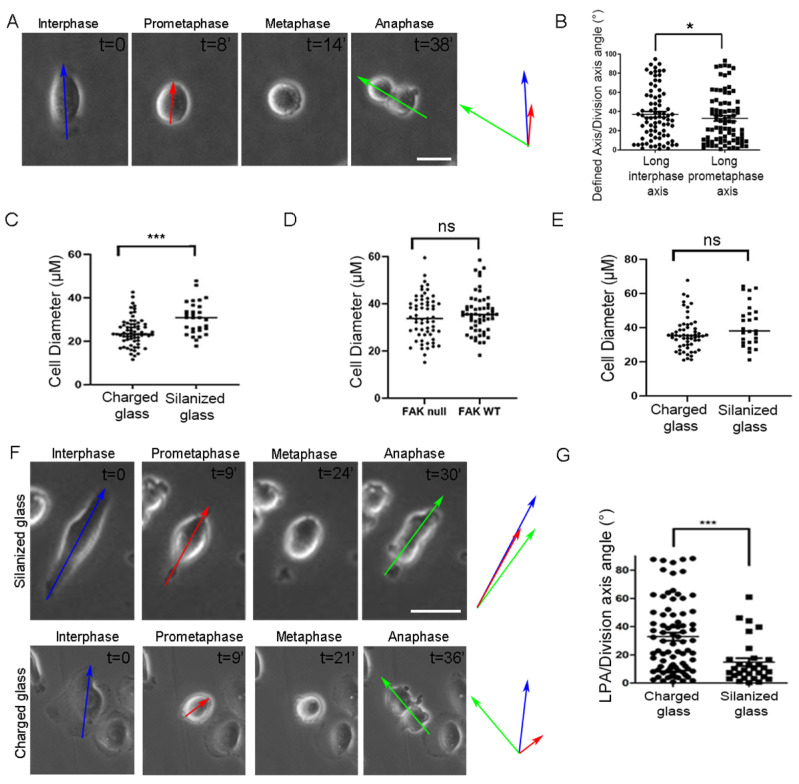
Increased substrate density rescues SO defects in FAK null cells. (**A**) Series of still images from a live recording of a misoriented mitotic FAK-/- cell seeded on charged glass with a significant difference between the division plane and the long interphase/long prometaphase axis. Blue arrow: long interphase axis, red arrow: long prometaphase axis, green arrow: division axis. t = time in minutes. Scale bar = 10 μm. (**B**) Graph comparing the angle between the long interphase axis/division axis and the long prometaphase axis/division axis in FAK-/- cells. The angle measured indicates the degrees of difference between each axis with respect to the division plane. Means ± SEM: Long interphase axis: 37.04 ± 2.966 (*n* = 80), long prometaphase axis: 32.87 ± 2.881 (*n* = 82). Statistical analysis was carried out using a paired *t*-test, *p* = 0.0353 (*). *n*, number of cells measured from three independent experiments. (**C**) Graph comparing the cell diameter (μΜ) of FAK-/- cells on charged vs. on silanized glass. Means ± SEM: Charged glass: 24.39 ± 0.7856 (*n* = 67), silanized glass: 30.65 ± 1.359 (*n* = 30). Statistical analysis was carried out using a Mann–Whitney test, *p* = 0.0002 (***). *n*, number of cells measured from three independent experiments. (**D**) Graph comparing the cell diameter (μΜ) between FAK-/- cells and FAK-/- cells + WT FAK on silanized glass. Means ± SEM: FAK-/- cells: 32.34 ± 1.191 (*n* = 61), FAK WT: 36.29 ± 1.181 (*n* = 58). Statistical analysis was carried out using a Mann–Whitney test, *p* = 0.3667 (ns), number of cells measured from three independent experiments. (**E**) Graph comparing the cell diameter (μΜ) of FAK-/- +WT FAK cells on charged vs. silanized glass. Means ± SEM: charged glass: 36.32 ± 1.323 (*n* = 58), silanized glass: 41.48 ± 2.408 (*n* = 27). Statistical analysis was carried out using a Mann-Whitney test, *p* = 0.0763 (ns), number of cells measured from three independent experiments. (**F**) (**Top panel**): Series of still images from a live recording of a mitotic FAK-/- cell seeded on silanized glass and the division plane corresponds to the long interphase and prometaphase axis. Blue arrow: long interphase axis, red arrow: long prometaphase axis, green arrow: division axis. t = time in minutes. Scale bar = 10 μm. (**Bottom panel**): Series of still images from a live recording of a mitotic FAK-/- cell seeded on charged glass and SO is random. t = time in minutes. Scale bar = 10 μm. (**G**) Graph comparing the angle between the long prometaphase axis/division axis in FAK-/- cells on charged and on silanized glass. Means ± SEM: Charged glass: 32.87 ± 2.881 (*n* = 82), silanized glass: 14.85 ± 2.814 (*n* = 30). Statistical analysis was carried out using a Mann–Whitney test, *p* = 0.0003 (***). *n*, number of cells measured from three independent experiments.

The fact that adhesion strength distribution can impact SO significantly suggests that cell types that fail to orient their spindle along the long interphase axis may simply display irregular adhesion strength distribution. Previous work suggested that a mechanosensory complex, established on integrin β1 and leading to the recruitment of FA proteins, including FAK, p130Cas, and Src, is formed on the lateral cortex of mitotic cells and controls spindle responses to external mechanical stimuli [23]. The members of this complex are involved in SO regulation, both in the z and the xy axes. Specifically, FAK-/- mouse embryonic fibroblasts display SO defects both with respect to the z-axis when attached to planar substrates but also fail to respond to adhesive micropatterns in the xy plane [11,23]. Although several lines of evidence suggest that FAK’s role in SO stems from its participation in the CMC, FAK null cells display defective cell/ECM interactions, which cannot be ruled out as the cause of the observed SO defects. Specifically, given that focal adhesion assembly and disassembly are both defective in FAK nulls, it is possible that a larger subset of these cells display an interphase/prometaphase discrepancy, which may be responsible for the observed orientation defects. We, thus, examined if the long prometaphase axis can better predict SO in FAK null cells. However, as shown, FAK nulls display misorientation both with respect to the long interphase and the long prometaphase axis (Figure 3A,B). These results are consistent with the notion that FAK is required for SO responses to external mechanical stimuli; however, they do not exclude the possibility that defects in cell adhesion lead to reduced force application on the cortex during metaphase, leading to SO defects.

### 2.3. Increased Substrate Density Rescues FAK SO Defects

FAK null cells display various adhesion defects, including delayed spreading, slower migration rates, and inability to reorganize adhesions in response to force application, among others [25,26,27]. In addition, FAK null cells display limited spreading and weak adhesion, which could lead to shorter and lower-density RFs, which in turn may not provide sufficient force on the cortex [28]. We, thus, decided to ask if providing substrates of increased density and strengthening cell adhesion may rescue SO defects in these cells. We achieved increased ligand density by plasma treating glass followed by amino silanization using APTES, which drastically increases protein adsorption [29]. Cells on amino silanized surfaces exhibited increased spreading compared to those on charged glass (Figure 3C). The spreading defect observed on glass was eliminated on silanized surfaces, with FAK nulls spreading to a similar extent as their rescued counterparts on glass (Figure 3D,E). Quantification of SO on silanized substrates revealed that the SO defects of FAK nulls were rescued on high-density substrates (Figure 3F,G), raising the possibility that FAK nulls display SO defects due to limited cell spreading or poor adhesion. Limited cell spreading would inadvertently lead to shorter RFs, which could potentially fail to exert sufficient force on the cell cortex to align the spindle.

**Figure 4:**
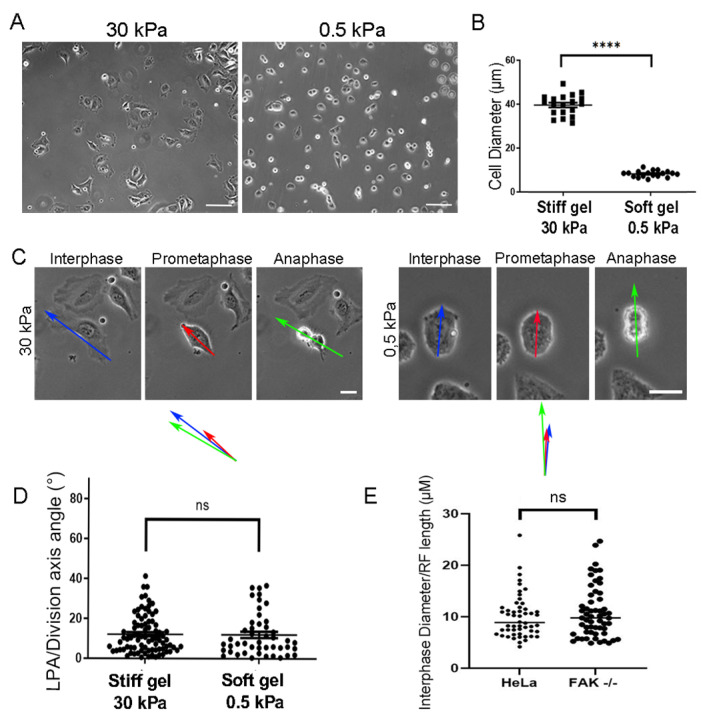
Limited cell spreading in HeLa cells is sufficient for proper SO. (**A**) Representative phase contrast images of cells seeded on 30 kPa and 0.5 kPa polyacrylamide gels. Scale bars = 50 μm. (**B**) Graph showing the cell diameter of HeLa cells on 30 kPa and 0.5 kPa polyacrylamide gels. Means ± SEM: Stiff gel 30 kPa: 39.49 ± 1.087 (*n* = 19). Soft gel 0.5 kPa: 8.228 ± 0.3399 (*n* = 18). Statistical analysis was carried out using an unpaired *t*-test, *p* ≤ 0.0001 (****). *n*, number of cells measured from two independent experiments. (**C**) Series of still images from live recordings of a mitotic HeLa cell with increased spreading on 30 kPa (**right**) and a Hela cell with limited spreading on 0.5 kPa polyacrylamide gel (**left**) and Scale bars = 10 μm. Blue arrow: long interphase axis, red arrow: long prometaphase axis, green arrow: division axis. (**D**) Graph comparing long prometaphase axis/division axis angles on stiff (30 kPa) and soft (0.5 kPa) gels. No significant difference is detected between conditions. Means ± SEM: Stiff gel 30 kPa: 12.07 ± 1.047 (*n* = 83). Soft gel 0.5 kPa: 11.90 ± 1.578 (*n* = 46). Statistical analysis was carried out using a Mann–Whitney test, *p* = 0.6566 (ns), number of cells measured from three independent experiments. (**E**) Graph comparing the ratio between interphase diameter (μΜ) to RF length (μM) between HeLa cells and FAK-/- cells. Means ± SEM: HeLa cells: 10.03 ± 0.5829 (*n* = 52), FAK-/- cells: 10.86 ± 0.6594 (*n* = 57). Statistical analysis was carried out using a Mann–Whitney test, *p* = 0.5679 (ns). *n*, number of cells measured from three independent experiments.

We, thus, turned to Hela cells and asked if limiting cell spreading would elicit SO defects. Substrate stiffness has a dramatic impact on cellular morphology, contractility, and spreading, with cells spreading more and becoming flat on stiff substrates and having limited spreading, weaker adhesion, and round morphology on soft substrates [30,31,32]. To manipulate cell spreading, we generated soft and stiff polyacrylamide gels, functionalized them, and proceeded to introduce cells and allow them to attach and spread [33]. As shown, cells on soft polyacrylamide gels (0.5kPa) exhibited drastically reduced spreading compared to the stiff polyacrylamide gel (30kPa) (Figure 4A,B). The average cell diameter was reduced by about 75%; however, despite the drastically reduced cell spreading on soft gels, spindle orientation was unaffected (Figure 4C,D). These data suggest that FAK-expressing cells can orient their spindle within the plane of adhesion accurately, even when cell spreading is drastically reduced and cell adhesion is weak. It also suggests that the limited cell spreading and weakened adhesion displayed by FAK null cells are not responsible for the SO defects of FAK null cells This is also consistent with our observation that the RFs are proportionally similar relative to cell size in both HeLa and FAK-/- cells on stiff substrates (Figure 4E).

### 2.4. SO Is Governed by Parallel Acting Mechanisms

**Figure 5:**
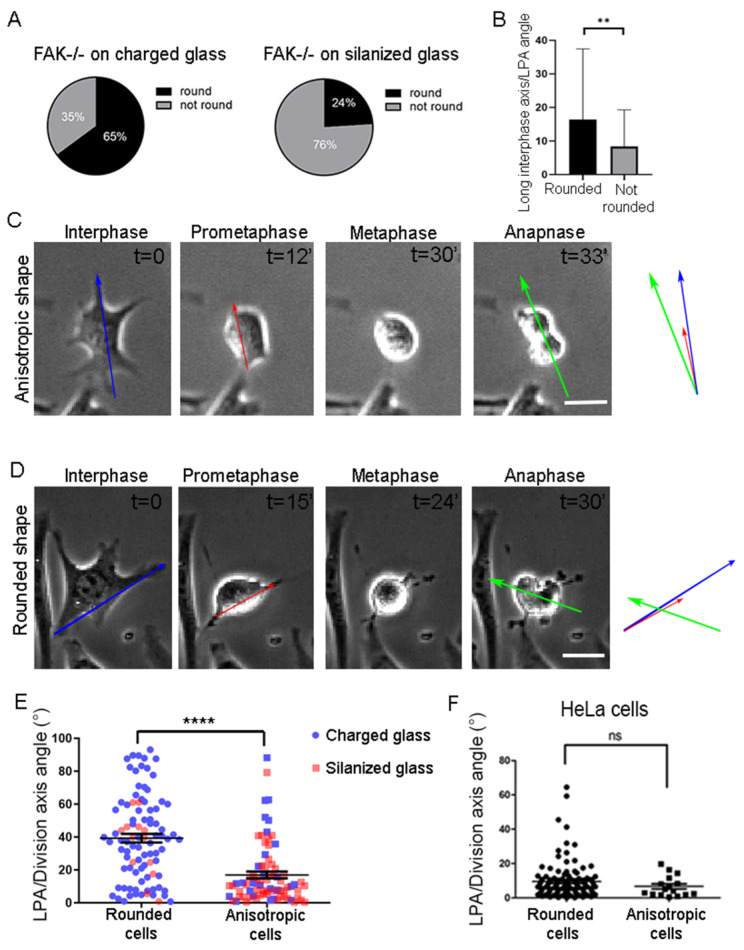
SO is defective in FAK-/- cells with full metaphase rounding. (**A**) Pie charts of the percentage of round vs. not round cells during prometaphase of FAK-/- cells on charged glass (*n* = 76) and on silanized glass (*n* = 30). (**B**) Graph comparing the long interphase to long prometaphase angle between round and not round mitotic FAK-/- cells. Means ± SEM: Round cells: 16.40 ± 2.485 (*n* = 72), not round cells: 8.381 ± 1.317 (*n* = 69). Statistical analysis was carried out using a Mann–Whitney test, *p* = 0.0050 (**). *n*, number of cells measured from three independent experiments. (**C**) Series of still images from a live recording of a mitotic FAK-/- cell that retains shape anisotropy during metaphase, and the division plane corresponds to the long interphase and prometaphase axis. Blue arrow: long interphase axis, red arrow: long prometaphase axis, green arrow: division axis. t = time in minutes. Scale bar = 10 μm. (**D**) Series of still images from a live recording of a mitotic FAK-/- cell that becomes fully round during metaphase, and SO is random. t = time in minutes. Scale bar = 10 μm. (**E**) Graph comparing the angle between the long prometaphase axis/division axis in FAK-/- cells that became round during metaphase and FAK-/- cells that retained shape anisotropy during metaphase. Colors represent the different conditions (blue = cells seeded on charged glass, red = cells seeded on silanized glass). Means ± SEM: Round cells: 39.33 ± 2.655 (*n* = 93), not round cells: 16.95 ± 2.079 (*n* = 78). Statistical analysis was carried out using a Mann–Whitney test, *p* ≤ 0.0001(****). *n*, number of cells measured from three independent experiments. (**F**) Graph comparing the angle between the long prometaphase axis/division axis in HeLa cells that became round during metaphase or that retained shape anisotropy during metaphase. Means ± SEM: Round cells: 9.471 ± 1.033 (*n* = 110), anisotropic cells: 6.688 ± 1.490 (*n* = 16). Statistical analysis was carried out using a Mann–Whitney test, *p* = 0.3285 (ns). *n*, number of cells measured from three independent experiments.

The above data show that high ligand density substrates can rescue SO defects presented by FAK null fibroblasts; however, experiments in FAK-expressing cells suggest that neither limited spreading nor weak adhesion can elicit SO defects [11]. To determine the underlying mechanism responsible for SO rescue on high-density substrates, we went on to track cell shape over time. We observed that a large percentage of cells failed to fully round up on salinized glass, even during metaphase, while the majority of cells fully round up on charged glass (Figure 5A). This analysis also revealed that in cells failing to fully round up, the long interphase and long prometaphase axis matched (Figure 5B). When cells from both substrates were grouped based on whether they achieved complete metaphase rounding, it became clear that only cells that failed to fully round up and retained a metaphase long axis (aspect ratio above 1.2) were oriented (Figure 5C,E), while cells that achieved complete metaphase rounding (aspect ratio under 1.2) failed to orient their spindle even on silanized substrates (Figure 5D,E) [34]. Thus, FAK null cells, unlike Hela cells (Figure 5F), fail to align their spindle along the interphase or prometaphase long axis if the cell accomplishes complete metaphase rounding.

The above suggests that in the absence of FAK, cells are unable to sense RF-derived forces when shape anisotropy is lost during metaphase. On the contrary, when they remain anisotropic, a shape-sensing mechanism drives SO, independent of FAK. This shows that cells have two independent mechanisms acting in parallel through which they determine SO in space, one based on shape sensing and a second based on cortical force sensing.

**Figure 6:**
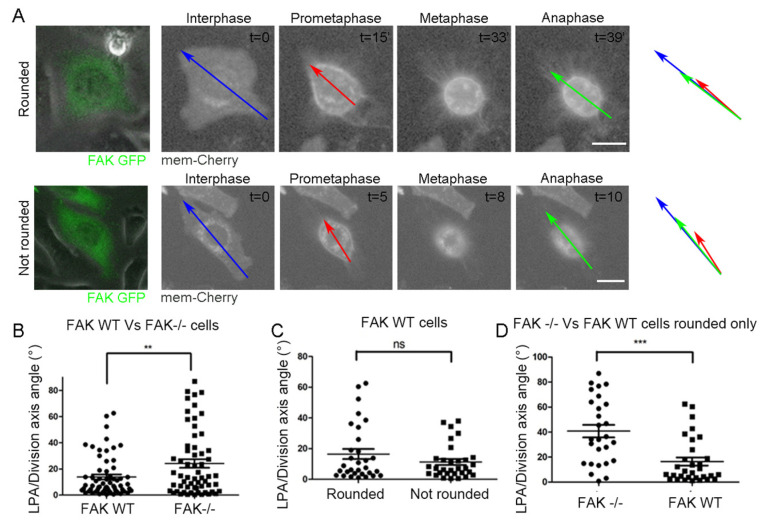
Introduction of FAK WT in FAK-/- cells rescues the defect of round mitotic cells. (**A**) Series of still images from live recordings of mitotic FAK-/- cells expressing FAK-GFP and mem-Cherry. Top row: a FAK-/- cell expressing FAK WT that becomes fully round during metaphase, bottom row: a FAK-/- cell expressing FAK WT that retains shape anisotropy during metaphase. mem-Cherry allows the visualization of the RFs. Blue arrow: long interphase axis, red arrow: long prometaphase axis, green arrow: division axis. t = time in minutes. Scale bar = 10 μm. (**B**) Scatter plot comparing the angle between the long prometaphase axis/division axis in FAK-/- cells expressing FAK WT and control FAK-/- cells. Means ± SEM: FAK-/- cells with FAK WT: 13.86 ± 1.930 (*n* = 62), FAK-/- cells: 24.12 ± 3.211 (*n* = 60). Statistical analysis was carried out using an unpaired *t*-test, *p* ≤ 0.006 (**). *n*, number of cells measured from three independent experiments. Scale bar = 10 μΜ (**C**) Scatter plot of the angles of the long prometaphase axis/division axis between FAK-/- cells expressing FAK WT that became round during metaphase and that retained anisotropy during metaphase. Means ± SEM: FAK WT round: 16.46 ± 3.289 (*n* = 31), FAK WT not round: 11.27 ± 1.970 (*n* = 31). Statistical analysis was carried out using a Mann–Whitney test, *p* = 0.5854 (ns). *n*, number of cells measured from three independent experiments. (**D**) Scatter plot of the angles of the long prometaphase axis/division axis between FAK-/- cells and FAK-/- cells expressing FAK WT that both became round during metaphase. Means ± SEM: FAK-/- cells: 40.75 ± 5.062 (*n* = 27), FAK-/- cells with FAK WT: 16.46 ± 3.289 (*n* = 31). Statistical analysis was carried out using a Mann–Whitney test, *p* = 0.0001 (***). *n*, number of cells measured from three independent experiments.

**Figure 7:**
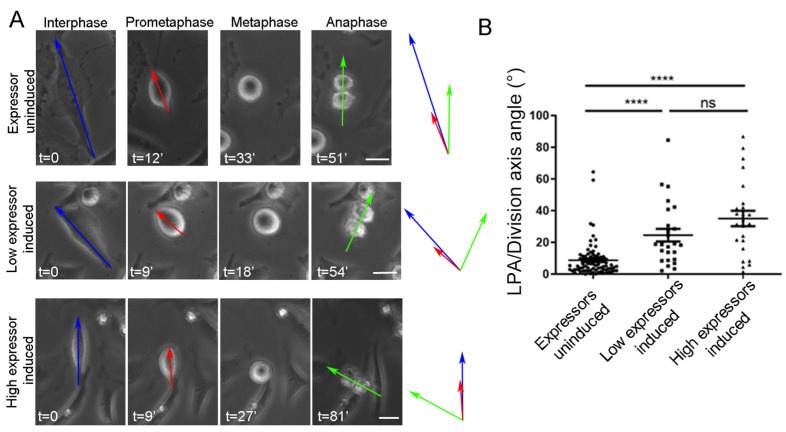
Expression of the FAK dominant negative LD2-LD4 in HeLa cells causes spindle misorientation. (**A**) Series of still images from live recordings of mitotic stable HeLa cells expressing LD2-LD4. Top row: no LD2-LD4 expression (expressor uninduced), middle row: low LD2-LD4 expression (low expressor induced), bottom row: high LD2-LD4 expression (high expressor induced). Blue arrow: long interphase axis, red arrow: long prometaphase axis, green arrow: division axis. t = time in minutes. Scale bars = 10 μm. (**B**) Scatter plot comparing the angles of the long prometaphase axis/division axis between no expression, low expression, and high expression of LD2-LD4 in HeLa cells. Means ± SEM: Expressors uninduced: 8.700 ± 1.179 (*n* = 82), low expressors induced: 24.56 ± 3.992 (*n* = 25), high expressors induced: 35.08 ± 4.858 (*n* = 24). Statistical analysis was carried out using a Mann–Whitney test, *p* ≤ 0.0001 (****) and *p* = 0.1079 (ns). *n*, number of cells measured from three independent experiments.

To validate this, we re-introduced WT FAK in FAK-/- cells. mCherry-mem was also introduced in the cells in order to confirm the formation of RFs in both round and anisotropic cells. As shown, the expression of WT FAK rescued the defect in round metaphase cells (Figure 6A–D), confirming that FAK has a crucial role in the determination of SO in the subset of cells that complete metaphase rounding via the sensing of RF-generated forces. These results were also confirmed through FAK inhibition in Hela cells. FAK inhibition was carried out using an inducible FAK inhibitory peptide, LD2-LD4, which we have shown to mask paxillin binding on the FAT domain, leading to loss of the paxillin FAK interaction previously shown to be essential for FAK role in SO responses to external mechanical stimuli [35]. HeLa cells are oriented correctly when complete metaphase rounding is achieved (Figure 5F). In addition, shape anisotropy retention during metaphase was a rare event in HeLa cells, which established a more robust cortical actin network during mitosis compared to fibroblasts [36,37]. Spindle angles with respect to the long prometaphase axis were calculated in control, low LD2-LD4, and high LD2-LD4 expressing cells. As shown, there is a dose-dependent defect in SO promoted by the expression of GFP-LD2-LD4 (Figure 7), showing clearly that FAK is required for the regulation of SO in HeLa cells that achieve full metaphase rounding.

**Figure 8:**
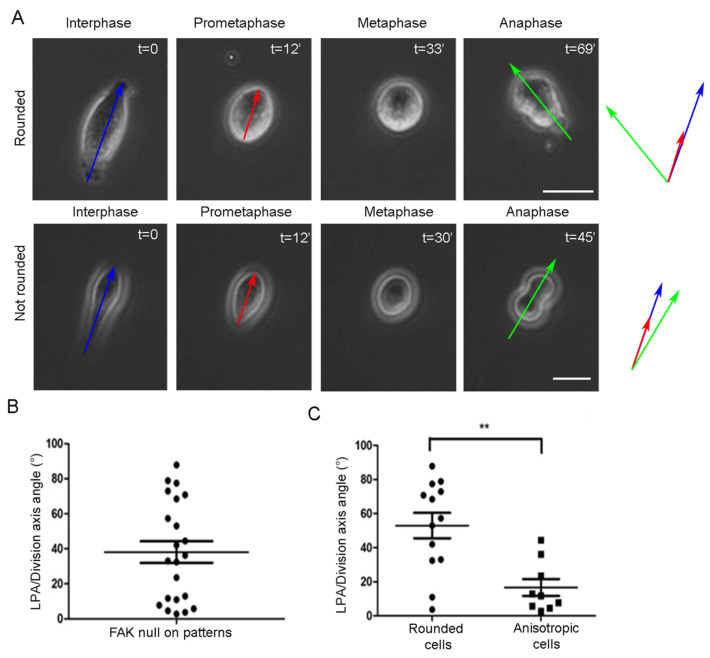
SO defects in FAK-/- cells are not a consequence of irregular adhesion strength distribution. (**A**) Series of still images from live recordings of mitotic FAK-/- cells seeded linear micro-patterned substrates. (**Top row**): a FAK-/- cell that becomes fully round during metaphase, (**bottom row**): a FAK-/- cell that retains shape anisotropy during metaphase. t = time in minutes. Blue arrow: long interphase axis, red arrow: long prometaphase axis, green arrow: division axis. Scale bars = 10 μm. (**B**) Scatter plot showing the angle between the long prometaphase axis/division axis in FAK-/- cells seeded on linear micro-patterns. Means ± SEM: 38.13 ± 6.136 (*n* = 22). (**C**) Scatter plot comparing the angle between the long prometaphase axis/division axis in FAK-/- cells that became round during metaphase and FAK-/- cells that retained shape anisotropy during metaphase on linear micro-patterned substrates. Means ± SEM: Round cells: 53.00 ± 7.423 (*n* = 13), not round cells: 16.65 ± 4.966 (*n* = 9). Statistical analysis was carried out using a Mann–Whitney test, *p* ≤ 0.0056 (**). *n*, number of cells measured from two independent experiments.

Although our data suggest that FAK reintroduction rescues SO through force sensing, we could not exclude the possibility that it instead restores a normal adhesion strength distribution pattern, which may indirectly lead to SO rescue. To exclude this possibility, we allowed FAK-/- cells to spread on highly anisotropic linear micropatterns, on which interphase shape anisotropy is increased, and adhesion strength distribution is identical and restricted along the micropattern for all cells. As shown, FAK nulls that achieve full metaphase rounding on micropatterned linear substrates are misoriented, while cells retaining shape anisotropy are oriented (Figure 8). This result confirms that the SO defects presented by FAK nulls stem from the inability to sense RF-derived forces and are not a consequence of irregular adhesion strength distribution.

Collectively, our data demonstrate that FAK is essential for correct SO in cells that achieve complete metaphase rounding, whereas it is dispensable in cells that retain shape anisotropy throughout mitosis. These results support the existence of two parallel mechanisms governing division plane determination: one mechanism relies on shape sensing to guide the spindle along the cell’s long axis when geometrical cues are maintained, while the other, driven by FAK-dependent force sensing, takes over in fully rounded cells by aligning the spindle with the axis of greatest force. Together, these complementary pathways ensure robust and accurate SO under diverse cellular conditions.

## 3. Discussion

Our study provides novel insights into the mechanisms governing spindle orientation in mammalian cells, specifically the involvement of cell shape and force, underscoring the complex mechanisms cells use to establish division planes. SO is fundamental to ensuring accurate genetic segregation, proper daughter cell positioning, maintaining tissue integrity and tissue morphogenesis. Hertwig’s original hypothesis proposed that thespindle orients along the long axis to divide the cell equally. O’Connell and Wang, as well as Minc et al., provided evidence supporting this hypothesis and suggested a shape-sensing mechanism involving astral microtubules and dynein [17,18]. Specifically, they suggested that the longer the astral MTs are, the more the motor protein dynein concentrates at their ends, exerting pulling forces on them. Given that the longer astral MTs are found along the long axis of the cell, they exert stronger forces that align the spindle along that axis.

In addition to cell shape, external forces have also been shown to impact SO. The Bornens group demonstrated that interphase adhesion geometry can accurately predict SO using micropatterned surfaces that confine cells to specific shapes [20,21,38]. Fink et al. subsequently proposed that mechanical forces can directly impact SO [22]. Specifically, external constraints on cell adhesion geometry create anisotropic RF force distributions that align the spindle along the axis of greatest force. This work highlights how external mechanical cues, when coupled with the cytoskeleton, can direct SO and suggests that forces applied to the cell cortex can influence SO even if they do not appreciably impact cell shape. Further evidence for the role of externally applied force on SO has been generated both in vivo and in vitro. For example, external tensile forces applied to cells, such as stretching, can directly affect SO. Studies have demonstrated that external stretching of cells can align the mitotic spindle along the applied tension [1,14,22,39,40]. This effect is mediated by cytoskeletal structures like actin filaments and retraction fibers (RFs) that transmit forces to the cell cortex. Tension stabilizes focal adhesions, and the stronger the FAs are, the more force the RFs can exert during mitosis. The alignment of the spindle along the axis of the greatest force in adherent cells is likely a result of this mechanical feedback loop. However, similar responses have been documented in cells of the Xenopus outer skin epithelium, which do not form focal adhesions and are not in contact with the ECM. Laser ablation of cells in close proximity to mitotic cells creates a wound, and subsequent wound healing exerts pulling forces on the mitotic cell that reorient the spindle in a FAK-dependent manner [11]. On the other hand, external compression can also influence SO. Experiments in confined environments, where cells are mechanically squeezed, show that SO can adjust to ensure cell division occurs perpendicular to the compressive force [17,34]. This response is thought to maintain tissue integrity and prevent mechanical stress from distorting the overall tissue architecture during cell proliferation; however, it is not clear if the cell senses the compressive force or the SO response stems from changes in cell shape. Overall, there is clear evidence that both cell shape and externally applied forces impact SO. How this information is processed and how force and shape inputs are integrated by the cell to determine the final SO is unclear. Both pushing and pulling forces impact cell shape, which complicates uncoupling their input on SO.

Here, we show that although the spindle in HeLa cells typically aligns with the interphase long axis, a subset of cells deviates from this pattern. In these cells, we show that the last axis of anisotropy prior to full cell rounding (typically prometaphase) and the interphase long axis do not coincide. These cells show a clear preference for the long axis formed during prometaphase. This discrepancy, particularly when the prometaphase and interphase long axes differ by more than 15 degrees, highlights the importance of cell geometry during prometaphase in the determination of SO. Our findings suggest that prometaphase geometry offers a more accurate prediction of SO in all cells. If a cell displays a different long axis at prometaphase than the long axis during interphase, the rate of retraction along the last axis of anisotropy must be slower than the rate along the interphase long axis, suggesting stronger adhesive contacts along the prometaphase long axis. Thus, the prometaphase long axis may provide a more accurate representation of the final force distribution around the mitotic cortex than the interphase axis, potentially due to differential focal adhesion (FA) strength or number, which influences retraction fiber (RF) density and distribution. Work by Matsumura et al. suggested that SO in adherent cells correlates well with the speed of retraction [41]. As a cell enters mitosis, it initiates membrane retraction alongside cell rounding. This involves a decrease in cell/ECM adhesion, driven by the disassembly of focal adhesions, and a concurrent increase in actomyosin contractility, which pulls the cell membrane inward [42,43,44]. In most cells, the long interphase and prometaphase axes match, and thus, rapid retraction rates do correlate with SO. However, in the subset of cells displaying a discrepancy in this regard, the spindle will instead align with the axis that displays slower retraction. These data suggest that SO in isotropic cells does not rely directly on edge retraction rates or a memory of the interphase adhesion geometry but is driven by force exertion on the cortex determined by the length and density of the retraction fibers.

We go on to further explore the roles of cell shape and force sensing, taking advantage of FAK null cells previously shown to fail to reorient their spindle in response to external mechanical cues both in vitro and in vivo. Our results confirm that FAK is required for SO responses to external mechanical stimuli, but it is dispensable when cells retain shape anisotropy. Interestingly, when cultured on high-density substrates, we noted that FAK null cells displayed improved adhesion and subsequent rescue of SO defects, suggesting that SO defects of FAK nulls may be a consequence of defects in adhesion. However, on substrates with limited cell spreading, SO defects re-emerged in FAK null cells but were absent in FAK-expressing cells, suggesting that weak adhesion and limited spreading are not responsible for the SO defects of FAK nulls. A more detailed analysis of FAK null cells on high ECM density substrates revealed that cells that orient their spindle correctly retain shape anisotropy throughout mitosis. Our data suggest that the force-sensing and shape-sensing mechanisms operate in parallel to regulate SO. We propose that cells rely on shape sensing when metaphase anisotropy is retained but shift to force sensing, dependent on FAK, when the cell undergoes complete rounding. This dual mechanism provides redundancy, ensuring correct SO even if one pathway is compromised. This could represent a finely tuned cellular strategy to accommodate variable adhesion environments, leveraging shape-sensing mechanisms when physical anisotropy restricts spindle alignment while utilizing FAK-dependent force sensing when shape constraints are absent. Our observations agree with previous studies in Xenopus, where SO persisted even in the absence of FAK under high anisotropy conditions, indicating that shape sensing alone can suffice under specific contexts [11]. They are also in agreement with work showing that in vertebrate epithelial tissues, where cells do not fully round up, force does not appear to impact SO directly or simply fine-tune this orientation, which is best predicted by the cell geometry [39].

While our study focuses on xy plane orientation, the mechanisms we describe are likely to operate similarly in 3D contexts where cell rounding and mechanical inputs vary across axes. Previous work has demonstrated that in mesenchymal cells, RF-driven forces maintain the spindle parallel to the substrate [11]. Together, these findings suggest that cells deploy a FAK-dependent mechanotransduction system to interpret force cues in both the planar and apicobasal planes, ensuring correct spindle orientation under a wide range of conditions.

Our study provides new insights into the mechanisms governing SO in mammalian cells. In summary, our findings suggest that SO is governed by two independent yet complementary pathways. When cell shape anisotropy is present, SO aligns with the long cell axis, potentially guided by astral MT-based length-dependent pulling forces. In contrast, when cells achieve complete metaphase rounding, SO relies on a FAK-mediated mechanism that detects force distribution around the cell cortex. This dual mechanism model provides an adaptable approach for cells to maintain robust control over SO, which is crucial for ensuring faithful division in diverse physical and mechanical contexts.

## 4. Materials and Methods

### 4.1. Cell Culture and Transfection

HeLa cell line and NIH3T3 cell line (ATCC) were cultured at 37 °C with 5% CO_2_ in DMEM with 10% FBS. FAK-/- cells were cultured at 37 °C with 5% CO_2_ in DMEM with 10% FBS and 1 mM sodium pyruvate. Transfections in HeLa and FAK-/- cells were performed with the use of Lipofectamine 2000 (11668019, Invitrogen, Carlsbad, CA, USA) according to the manufacturer’s protocol. Plasmids used were purchased from AddGene (Watertown, MA, USA).

### 4.2. Silanization of Coverslips

Glass coverslips were initially cleaned in a sonicator bath for 15 min. After washing and drying, the coverslips were then exposed to plasma for 5 min for charging. Silanization was performed with the use of (3-Aminopropyl) triethoxy-silane (Alfa Aesar, Heysham, Lancashire, UK) diluted in isopropanol to a final percentage of 20%. Coverslips were incubated in the diluted solutions for 90 min, followed by three isopropanol washes and baking at 100 °C for 30 min. All incubations were performed in sealed chambers. Coverslips were either used immediately or stored in a sealed container for use for up to one week.

### 4.3. Substrate Coatings

Charged or silanized coverslips were coated with bovine plasma Fibronectin (33010018, Thermo Fisher Scientific, Waltham, MA, USA) at a concentration of 10 μg/μL after incubation for 1 h at 37 °C, followed by two washes with 1X PBS before seeding of cells.

### 4.4. Generation of Different Stiffness Polyacrylamide Gels

For the generation of different stiffness polyacrylamide gels, we followed the information provided by the study of Syed et al. 2015 [33]. More specifically, the concentrations of acrylamide and crosslinker bis-acrylamide were used as described in Table 1 of Syed et al. 2015 [33], to generate gels of the corresponding stiffness. A total of 15 μL of polyacrylamide mix was placed between a silanized coverslip and a RainX coated coverslip (RainX coating was performed with immersion of coverslips in RainX for 10 s, followed by quick isopropanol washes and air drying). After allowing the gels to set for 1 h RT, we incubated them in water for 1 additional hour to allow efficient detachment of the acrylamide gel from the RainX coverslip. After the detachment of the RainX coverslip, Sulfo-SANPAH was added to the gel surface and exposed under UV light for 5 min to activate the crosslinker. After 3 1xPBS washes, coverslips with gels were incubated overnight with 10 μΜ Fibronectin. The following day, gels were equilibrated in DMEM for 1 h. After equilibration, they were ready to use for cell seeding.

### 4.5. Imaging

Live imaging for the acquisition of mitotic events was performed in an inverted Carl Zeiss Axiovert 200M Motorized Inverted Fluorescence Microscope (Zeiss, Oberkochen, Baden-Württemberg, Germany) with a temperature-controlled system ibidi Heating System Multiwell Plate-Silver Line (ibidi, Gräfelfing, Germany).

### 4.6. Statistical Analysis

AxioVision Rel 4.8 software was used for the calculation of spindle angles in live recordings. All measurements were analyzed with GraphPad Prism 8.

## Data Availability

Data is contained within the article.
